# Preventing the progression of text neck in a young man: A case report

**DOI:** 10.1016/j.radcr.2021.12.053

**Published:** 2022-01-18

**Authors:** Eric Chun-Pu Chu

**Affiliations:** New York Chiropractic and Physiotherapy Centre, 41/F Langham Place Office Tower, 8 Argyle Street, Hong Kong SAR, China

**Keywords:** Cervical radiculopathy, Cervical spondylosis, Chiropractic manipulation, Surface electromyology, Text neck

## Abstract

Text neck describes an overuse injury of the cervical spine resulting from the repetitive stress of prolonged forward head flexion while looking down on a mobile screen. This case report describes a 24-year-old young man who presented with a 12-month history of head and neck pain and paresthesia of the right upper limb. The patient worked as a YouTuber and has been editing and posting videos on the website for three years. One year prior to referral for chiropractic assessment, the patient first visited his family physician for similar complaints. Based on cervical radiographs, the diagnosis of cervical spondylosis was given. Previous management included pain medication and muscle relaxants. Interventions included repeated physical therapy, cervical traction, and acupuncture, with some temporary relief during the subsequent year. However, severe flare-up of the symptoms occurred, which was brought about by working for extended periods on his smartphone, for which the patient sought chiropractic attention. X-ray imaging showed cervical kyphosis with C5 vertebral rotation, hypertonicity of the paraspinal muscles, and paresthesia in the right C6 dermatome distribution, which were consistent with text neck syndrome associated with cervical spondylosis and right C6 radiculopathy. The intervention consisted of improving posture while texting, cervical manipulation, and extension traction therapy. After 9 months of treatment sessions, the symptomatic and functional improvement was reflected by the radiographic changes in the cervical curve correction and the normalized paraspinal muscle tension on surface electromyology. Frequent breaks along with correct posture while using smartphones will be the key entities to prevent the occurrence of text neck syndrome.

## Introduction

Text neck refers to the degeneration of the cervical spine resulting from the repetitive stress of prolonged forward head flexion while looking down at mobile screens [1]. If left insufficiently treated, the text neck can worsen over time, causing a multitude of physical health problems such as cervical curvature alteration, neck and shoulder muscle strain, impaired neck muscle perception, posterior ligamentous injury, and entrapment neuropathies [2]. Disorders associated with flexed head posture include cervicogenic headaches [3], cervicogenic dizziness [2,4], and cervical radiculopathy [5,6]. Most of these conditions manifest with clusters of painful symptoms and spine dysfunctions.

This case highlights the progression of text neck, which was insufficiently treated in a young man. The patient subsequently underwent nine months of chiropractic intervention. He reported resolution of pain and neurological symptoms in the neck and shoulder and regained neck mobility, which were supported by the improvement of radiographic and electromyological findings. Surgery is usually not indicated for mechanical causes of neck pain, unless there are progressive neurologic deficits or intractable pain and disability that is unresponsive to conservative treatments [2]. Avoiding prolonged texting along with maintaining a correct position are keys in preventing or addressing text neck [7].

## Case report

**Fig. 1:**
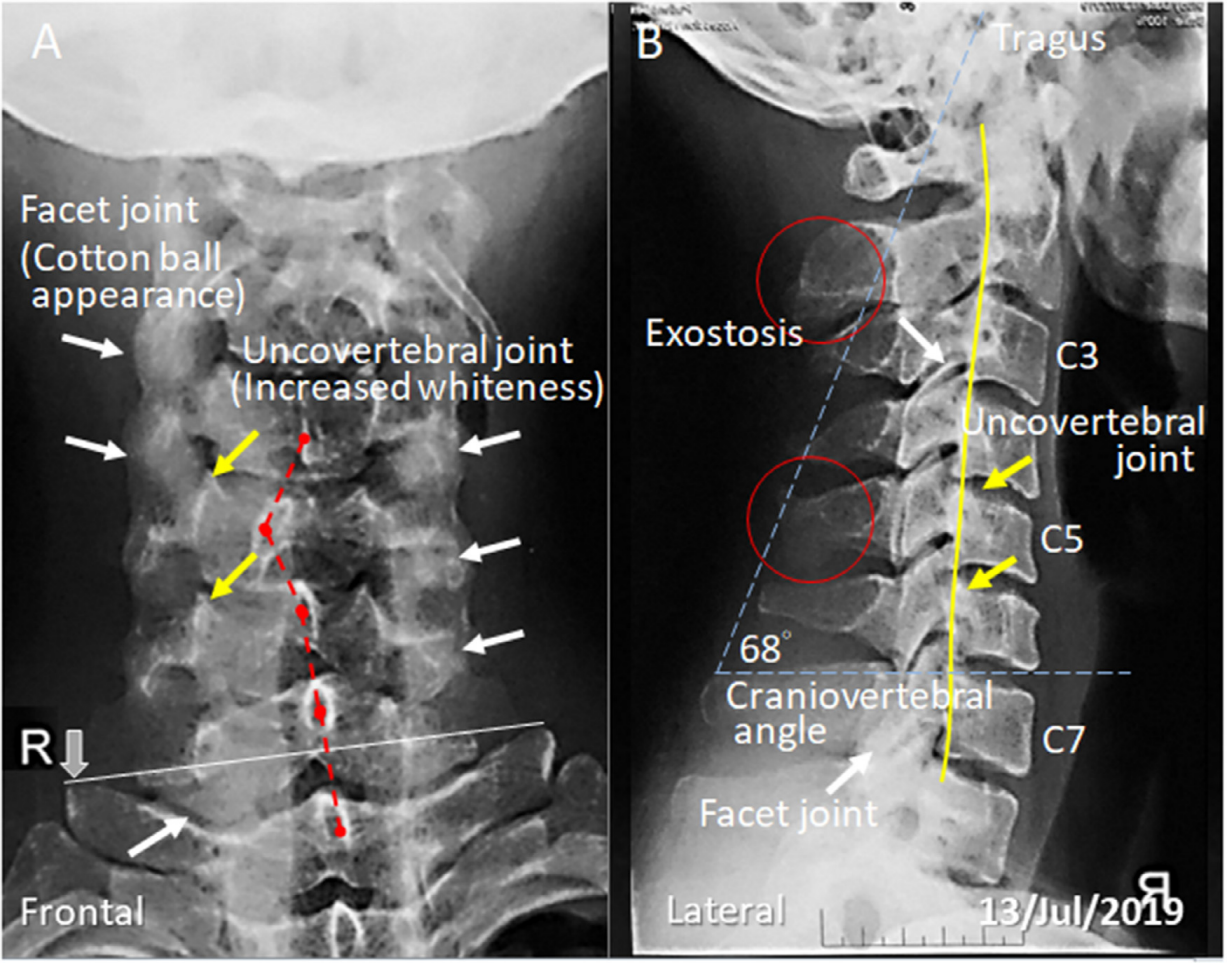
Cervical radiographs taken one year before chiropractic attention. (A) Frontal cervical X-ray shows a tilt to the right and degenerative hypertrophy of the facets (white arrows) and uncovertebral joint (yellow arrows). Dashed red line highlights malalignment of the C-5 spinous process, indicative of a vertebral rotation. (B) Lateral cervical x-rays revealed a loss of cervical lordosis (yellow line highlights posterior vertebral body margins). Global cervical curve is 0**°**, using the posterior vertebral body of C2 and the posterior vertebral body of C7. The exostoses (red circles) in the spinous process interspaces indicate a chronic injury of the nuchal osteo-ligamentous attachment. The craniovertebral angle is 68**°**. An angle less than 50**°** will be defined as forward head posture.

A 24-year-old male YouTuber presented with head and neck pain and paresthesia of the right upper limb for 12 months. He denied any injury event. During the past three years, the patient regularly edited blogging and videos on the YouTube website. He relied heavily, at least 16 hours a day, on a smartphone for both job resources and personal tasks. He would check his screen every 10 minutes. One year prior to this diagnosis, the patient experienced similar symptoms and visited his family physician. Cervical radiographs (Fig. 1) exhibited reduced cervical lordosis, axial rotation of the C5 vertebra, and degenerative proliferation of facets (white arrows) and uncovertebral joints (yellow arrows), suggestive of cervical spondylosis. Previous management included pain medication and muscle relaxants. Interventions included repeated physical therapy, cervical traction, and acupuncture, with some temporary relief. However, 12 months later, the patient repeatedly experienced severe flare-ups of the symptoms. This time, the patient could keep his head up for only a minute and was unable to move his neck without pain. These new difficulties forced him to seek chiropractic attention.

**Fig. 2:**
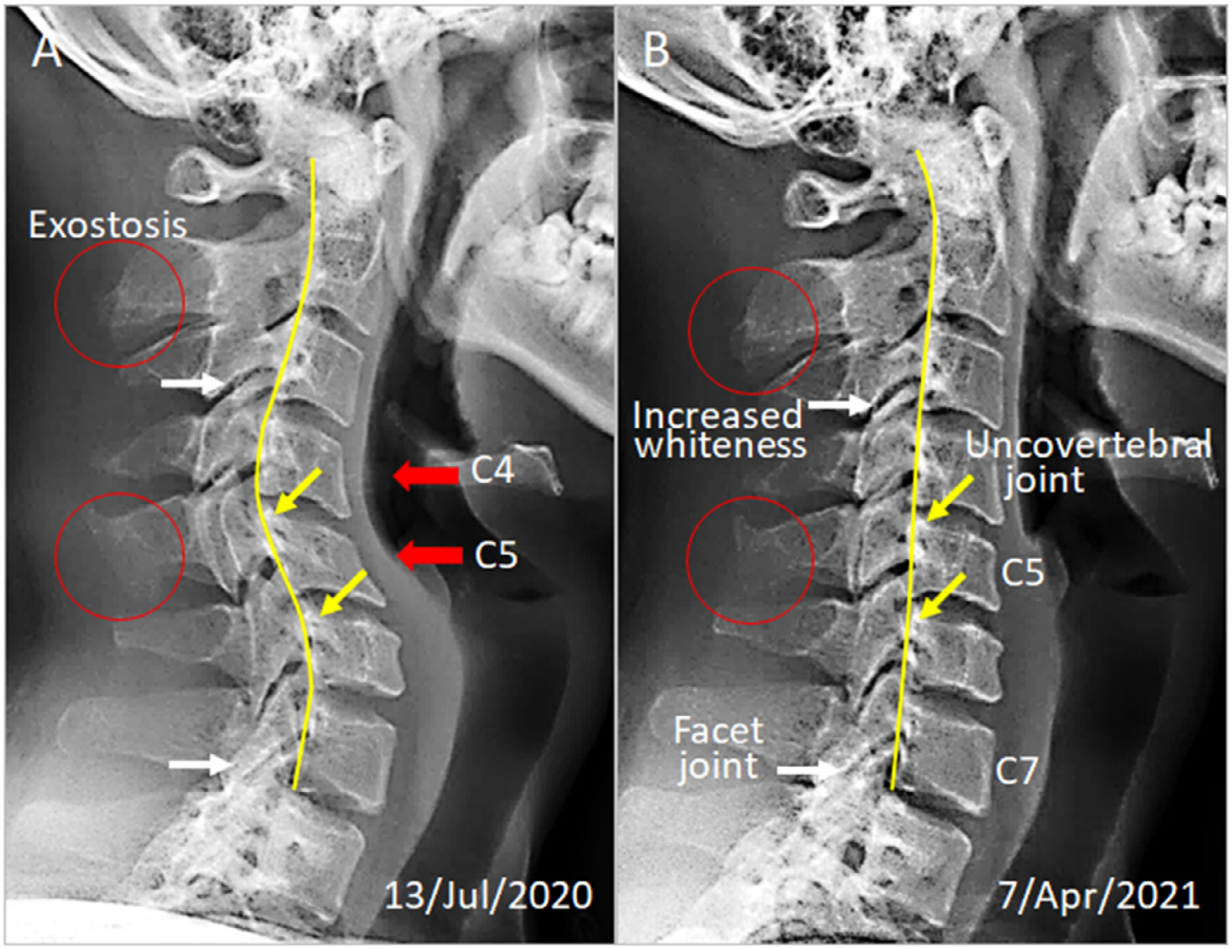
Comparison of cervical alignment on sagittal radiographs. (A) Pretreatment image depicts cervical kyphosis and subluxation of the C4 with relation to C5. Red arrows point to the two segments that are developing reversed curve. Arthritic cervical facet joints and uncovertebral joints are denoted by white and yellow arrows, respectively. Kyphotic angle of the C4/C5 is 23°. The posterior vertebral (yellow) line highlights a distorted curvature. (B) After nine months of treatment, the C5 vertebral rotation was restored, and global cervical curvature returned to 2° lordosis. The radiographic and postural improvement positively correlates with symptom relief.

**Fig. 3:**
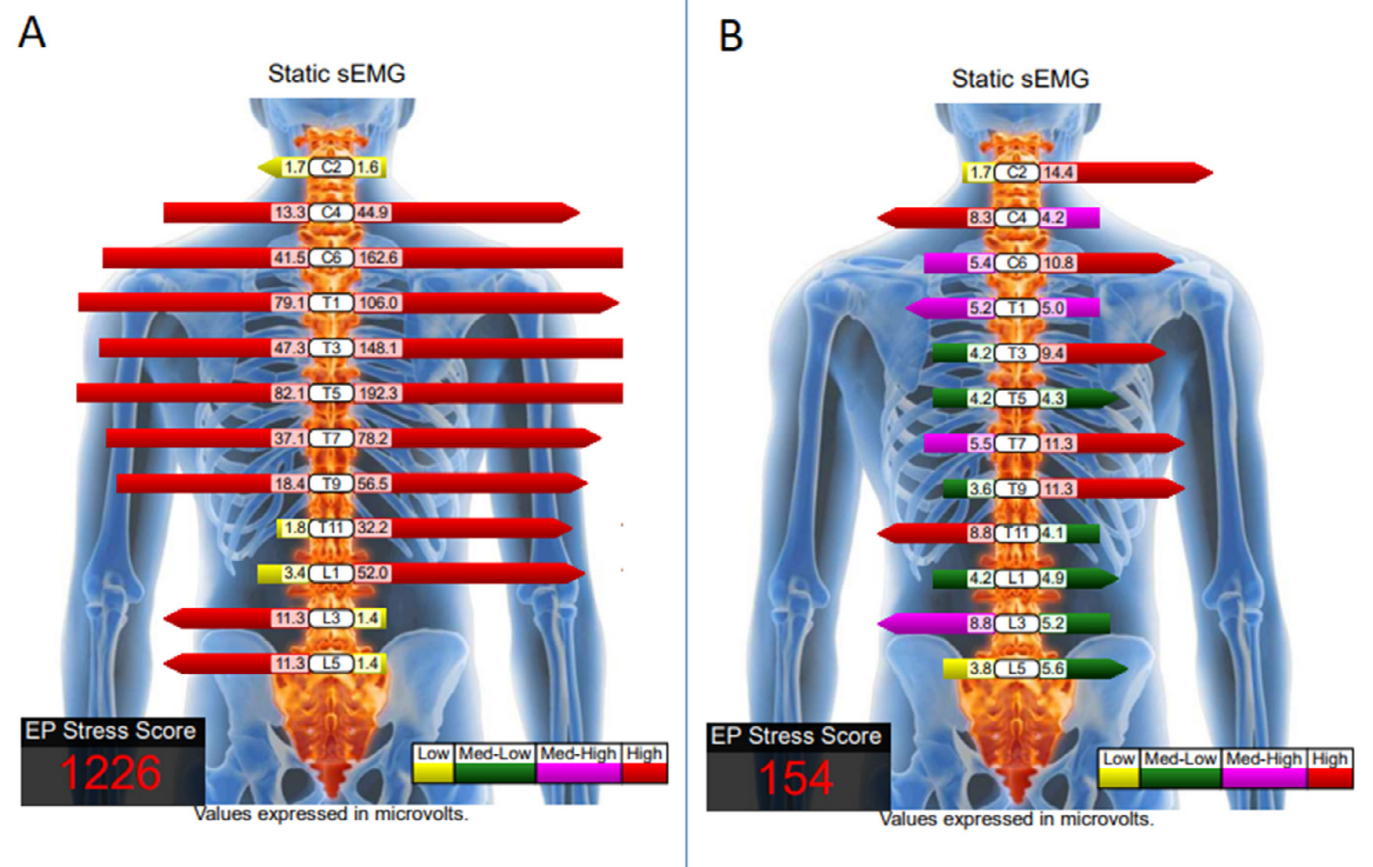
Surface electromyology (sEMG) comparison of pre- and post-intervention. A set of handheld electrodes is applied to the paraspinal muscle group for each measured level. The levels of muscle tension are expressed with the length and color of the bars in the schematic representation. The electrophysiological (EP) stress score is the sum of all muscle activity on the spine, measured in microvolts. (A) High readings reflect spasm in the neck and thoracic paraspinal muscles at initial presentation. (B) Normalized sEMG signal of the paraspinal muscles and dropped EP stress score are positively correlated with symptom relief after the nine-month chiropractic therapy. Mild muscle tension sustained in the right neck, shoulder, and mid-back.

Upon initial evaluations, the patient presented with a guarded neck posture. He stated that he suffered with head and neck pain and intermittent numbness from his right shoulder to lateral forearm and hand. The self-reported peak pain intensity of his neck pain and headache was 5/10 on an 11-point numeric pain rating scale [8]. The cervical range of motion was limited by pain to 30° extension (normal > 70°) and 50° at both rotations (normal > 90°). There was protective muscle spasm around the neck. Hypertonicity was palpated in the bilateral trapezius, rhomboid, sternocleidomastoid, and levator scapulae muscles. The C5/6, C6/7, T2/3, and T7/8 segments were restricted. Paresthesia distribution was consistent with the right C6 dermatome. Cervical radiographs (Fig. 2) depicted a reversed curvature, subluxation of C4 with relation to C5, and degenerative arthritis (arrows). Surface electromyology (sEMG) depicted high readings reflecting spasm in the neck and thoracic paraspinal muscles (Fig. 3). The patient was diagnosed with text neck syndrome associated with cervical spondylosis and right C6 radiculopathy.

The patient was educated to take frequent rests and raise his smartphone up to the eye level while texting. The therapeutic intervention began with a cervical manipulation to release muscle spasm and intervertebral restriction, restore cervical mobility, and mitigate neurological dysfunction. Treatment sessions were scheduled thrice a week for the first phase. His neck pain reduced by 60% after the first month, and all neurological symptoms were resolved within 2 months. The patient stopped all medications. During the next 4 months, a computerized extension traction therapy (iTrac Cervical Curve Remodeling System, SD, USA) was added to restore cervical curve and reduce instability. Gentle cervical traction was administered based on the preset force, angle, and time duration to get the desire therapeutic effect. Treatment frequency was then reduced to twice a week. The patient regained full cervical range of motion and was subsequently continued an elective spinal rehabilitation program, focusing on the maintenance of cervical alignment, twice a month during the next 3 months.

On the ninth month re-evaluation, the symptomatic and functional improvement was reflected by the radiographic changes of the cervical curve correction (Fig. 2B) and the normalized sEMG findings (Fig. 3B). No treatment-related adverse events were reported.

## Discussion

Mobile texting and gaming is a growing lifestyle and health concern, with the constant growth in mobile/smartphone usage and adoption. Researches [9,10] revealed that the distinct cervical flexion seen in heavy smartphone users is causing a new overuse condition known as "text neck." The fulcrum for flexion in the cervical spine in adults is at the C5/C6 level [2]. A neck flexion of 0° to 15° is akin to having the head squarely over the shoulders, wherein the stress on the cervical muscles is acceptably low [9]. A static and flexed neck posture can cause continuous strain of the posterior cervical musculature (the cervical erector spinae and suboccipital muscles, levator scapulae, and semispinalis and trapezius muscles), producing tension headaches, neck and shoulder pain, temporomandibular joint pain, and decreased cervical and upper thoracic ranges of motion [11]. Sustained flexed stress can also slacken posterior ligamentous structures, resulting in instability between vertebral segments, degenerative spondylosis, and vertebral body sliding [2,11]. In the long term, text neck can lead to plastic changes within the nervous system, causing sensorimotor integration deficiencies and further dysfunction [11].

When the neck is in flexion position, the paraspinal muscles act as antagonistic muscles to maintain compliance of the posture, which results in increased myoelectric activity [12]. Surface electromyography (sEMG) is a reliable technique to evaluate muscle activity. Applying two handheld recording electrodes on both sides of the spine 4 cm apart, static sEMG will record and process the level of muscle guarding quantitatively. The signal amplitude of sEMG is positively related to the amount of force produced by the muscles. The levels of muscle tension are displayed with the length and color of the bars in the schematic representation. The sum of all muscle activity readings (in microvolts) for both sides at all levels of the spine is referred to as the electrophysiological (EP) stress score (Fig. 3). In the present study, sEMG was used to assess the myoelectric activity of the paraspinal muscles in the patient with text neck syndrome before and after treatment. It was found that the signal amplitude and EP stress score were significantly different before and after the 9-month chiropractic therapy and were significantly correlated with symptom relief and cervical curvature correction, suggesting that sEMG could be used to objectively assess muscle functional change.

A long-lasting neck flexion due to excessive texting on a smartphone was assumed to be hazardous to cervical structures. Researchers have observed that 10 minutes of static flexion can lead to changes in mechanical and neuromuscular behavior of the cervical spine, potentially leading to decreased strength of cervical spine structures [13]. Surgery is usually not indicated for mechanical causes of neck pain, unless there are progressive neurologic deficits or intractable pain and disability that is unresponsive to conservative treatments [2]. Manual therapy has been shown to have a clinical benefit in correcting reversed cervical curvature [4,6,14]. Treatments for text neck syndrome should target neck problems and be tailored for individual patients based on their treatment responses. Regarding the current case, extension traction therapy is designed to target the anterior longitudinal ligament that attaches to the front of each vertebra. The viscoelastic properties of ligament fibers allow them to accommodate sustained loads. It is presumed that the restoration of natural cervical lordosis following extension traction therapy is mostly due to ligamentous creep (stretching) [15]. Text neck is an altogether preventable overuse degeneration that must be brought into high awareness for smartphone users [11].

In conclusion, the current study reported a progression of text neck, which has been insufficiently treated. Sustained flexion of the neck will cause cervical spine distortion. The improvement of neurologic symptoms has been shown to correlate with radiographic and electromyological alterations responding to the correction of cervical misalignment.

## Patient Consent

Written informed consent was obtained from the patient whose files were used in this study.
